# Younger adult type 2 diabetic patients have poorer glycaemic control: a cross-sectional study in a primary care setting in Singapore

**DOI:** 10.1186/1472-6823-13-18

**Published:** 2013-06-03

**Authors:** Joanne Hui Min Quah, Yan Ping Liu, Nan Luo, Choon How How, Ee Guan Tay

**Affiliations:** 1 SingHealth Polyclinics- Outram Polyclinic, 3 Second Hospital Avenue, Health Promotion Board building Level 2, Singapore 168937, Singapore; 2 Merck Sharp & Dohme (I.A.) Corp, (Singapore Branch), Singapore, Singapore; 3 Saw Swee Hock School of Public Health, National University of Singapore, Singapore, Singapore

**Keywords:** Diabetes mellitus type 2, Hemoglobin A glycosylated, Primary health care, Singapore

## Abstract

**Background:**

The aim was to study the glycaemic control of type 2 diabetic patients, and to identify factors associated with unacceptable glycaemic control (defined as HbA_1c_ >8.0%).

**Methods:**

Analysis of data collected in a cross-sectional survey of type 2 diabetic patients in eight SingHealth Polyclinics in January 2009. HbA_1c_ value was measured on the day of the survey, while information on patient and diabetic characteristics was obtained through a questionnaire. Odds ratio of having unacceptable glycaemic control was estimated for selected variables using multiple logistic regression models.

**Results:**

A total of 688 patients were included in the analysis. The mean (± standard deviation) and median (range) HbA_1c_ levels were 7.6% (± 1.35) and 7.3% (5.0% to 14.0%), respectively. 25.4% of the patients had an unacceptable HbA_1c_ level of >8.0% and the odds of this were higher (p < 0.05) in patients with the following characteristics: younger age, longer diabetes duration, presence of insulin treatment, and poorer compliance to medication.

**Conclusion:**

Younger adult patients were found to have poorer glycaemic control, and hence targeted educational and behaviour modification programmes would be required to effectively manage this group of patients.

## Background

Type 2 diabetes mellitus is now a major chronic disease epidemic. An estimated 285 million people worldwide were affected in 2010 [1], with 1.3 million deaths due to diabetes [2].

Type 2 diabetes mellitus causes significant mortality and morbidity [3] due to its long-term micro-vascular and macro-vascular complications, and these adverse outcomes are associated with poorer glycaemic control [4]. Each 1% reduction in updated mean HbA_1c_ has been shown to be associated with reduction in risk of 21% for deaths related to diabetes, 14% for myocardial infarction, and 37% for microvascular complications [5]. Despite clinical evidence of the beneficial effect of glycaemic control and the advances achieved in diabetes control and treatment, the management of type 2 diabetes mellitus remains challenging. Data from different regions of the world show that the majority of patients with type 2 diabetes mellitus are not controlled to the recommended HbA_1c_ level [6,7].

There is also increasing recognition that intensive (versus conventional) glucose lowering treatment has limited benefits on all-cause mortality and deaths from cardiovascular causes, and the harm associated with severe hypoglycemia might counter balance the potential benefit of intensive glucose lowering treatment [8]. This suggests that glucose-lowering regimens should be tailored to the individual patient.

With rapid urbanization development in the past few decades, Singapore has emerged a country with high prevalence of diabetes mellitus. From 2004 to 2010, the percentage of Singapore residents with diabetes aged 18 to 69 years old has increased from 8.2% to 11.3%. [9]. Diabetes was the 10th leading cause of death in 2009 and contributed to 1.7% of all deaths locally [10]. Using the Singapore Diabetes Mellitus Clinical Practice Guidelines [11], the HbA_1c_ value has been classified into four categories, ideal: 4.5% to 6.4%; optimal: 6.5% to 7.0%; suboptimal: 7.1% to 8%; and unacceptable: >8%. In our study, we used the cut off value of >8% to identify patients with unacceptable glycaemic control.

In Singapore, the majority of patients with type 2 diabetes mellitus are treated in the primary health care setting. SingHealth Polyclinics is a group of 9 public primary care polyclinics serving the central and eastern parts of Singapore. In 2010, SingHealth Polyclinics had a total of 1.746 million medical patient attendances, 7.7% of these attendances were for diabetes mellitus, and we currently have 55,000 active patients in our diabetes database.

Information on glycaemic control of the patients in the polyclinics is of great value for planning diabetes management programs to prevent and delay the onset of chronic complications. The aim of our study was to 1) investigate the prevalence of suboptimal glycaemic control and 2) identify factors associated with unacceptable glycaemic control in type 2 diabetes mellitus patients treated and followed up in SingHealth polyclinics.

## Methods

### Study design

We conducted a cross-sectional survey in eight SingHealth Polyclinics, including Bedok, Bukit Merah, Geylang, Outram, Pasir Ris, Queenstown, Sengkang, and Tampines Polyclinics, which are located in the residential areas in Singapore. Results are from the analysis of data collected by third year medical students from Yong Loo Lin School of Medicine, National University of Singapore conducting a Community Health Project in January 2009. The study objectives were to study glycaemic control, as well as health-related quality of life of type 2 diabetic patients treated in SingHealth Polyclinics which was previously reported [12].

Systematic sampling was used to recruit the participants. During the study period, recruiters were stationed at the clinical laboratory of the polyclinics, and all patients coming to the laboratory were approached. All type 2 diabetic patients above 21 years of age on follow-up at SingHealth Polyclinics who had the glycosylated haemoglobin (HbA_1c_) test on the day of survey as part of their routine 3-monthly visit, and who were able to communicate to give informed consent were identified. Every other eligible type 2 diabetic patient was invited to participate in the study.

An interviewer-administered questionnaire was conducted after written consent was obtained. Patients competent in the English, Chinese, or Malay were also given the option to self-administer the questionnaire survey. In the event that the patient was illiterate and was not able to communicate well with the interviewer, his or her care-giver would be the administrator. The patients were aware that they were free to terminate their participation at any point of time through the conduct of the survey. This study was approved by the Institutional Review Board of SingHealth Polyclinics.

### Statistical analysis

All statistical analyses were conducted using SPSS version 17.0. The HbA_1c_ value was the dependent variable in data analyses. The value of HbA_1c_ was taken from the test result and is a continuous variable, and analysed as a binary outcome (greater than 8% vs less than/equal to 8%). All other variables were categorical data, for example, age was coded as three groups: <60 years old, 60–69 years old, and 70 years and above. The statistical analyses included both descriptive and association analyses. The descriptive analysis was conducted for demographic, socio-economic and diabetic disease characteristics. Association analysis of possible predictors for unacceptable glycaemic control was conducted in two steps. First, bivariate logistic regression was performed for each variable to examine unadjusted association with unacceptable HbA_1c_ control; second, variables which were identified to be significantly (p < 0.05) associated with the unacceptable HbA_1c_ control in the bivariate analysis were included in a multivariate logistic regression model for assessing adjusted effects of predictors on poor HbA_1c_ control. Age, gender and ethnicity were also included in this multivariate logistic regression model as independent variables.

## Results

### Patient recruitment

Using a systematic sampling method, a total of 699 subjects participated in our study, representing a response rate of 72.5%. Of this, 688 had valid HbA_1c_ values and were included in the final data analysis.

### Distribution of HbA_1c_ level

The mean (±standard deviation) and median (range) HbA_1c_ level was 7.6% (±1.35) and 7.3% (5.0% to 14.0%), respectively. Using the Singapore Diabetes Mellitus Clinical Practice Guidelines 2006 [11], 25.4% of the patients had an unacceptable HbA_1c_ level of >8%, 39.3% fell into the suboptimal category with HbA1c level of 7.1-8.0%, 22.8% had an optimal HbA1c level of 6.5-7.0%, and 12.2% of patients had an ideal HbA1c level of 4.5-6.5%.

### Patient characteristics

Patient demographic characteristics are presented in Table 1. Among the patients recruited, the mean age was 62.2 (±11.1) years old and 64% of the patients were 60 years of age and above. Slightly more than half the patients were female (56.0%). Majority of patients were Chinese (73.3%). The percentage of Indian patients was 11.6%, disproportionally higher than the national level of 9% [13], reflecting the higher prevalence of diabetes mellitus in the ethnic Indian group.

**Table 1:** Patient demographic characteristics

**Characteristics**	**HbA1c ≤ 8.0%**	**HbA1c > 8%**	**Total**	**p-value**
	**(n = 513)**	**(n = 175)**	(n = 688)*	
	**N (%)**	**N (%)**	**N (%)**	
**Gender**				
Male	226 (44.2)	76 (43.4)	302 (44.0)	0.854
Female	285 (55.8)	99 (56.6)	384 (56.0)	
**Age, mean (standard deviation), year**	65. 0 (11.4)	58.3 (10.1)	62.2 (11.1)	<0.001
**Age group**				
<60 years	156 (30.7)	90 (51.4)	246 (36.0)	<0.001
60-69 years	187 (36.8)	49 (28.0)	236 (34.6)	
≥ 70 years	165 (32.5)	36 (20.6)	201 (29.4)	
**Ethnicity**				
Chinese	390 (76.0)	114 (65.1)	504 (73.3)	0.023
Malay	55 (10.7)	27 (15.4)	82 (11.9)	
Indian	51 (9.9)	29 (16.6)	80 (11.6)	
Others	17 (3.3)	5 (2.9)	22 (3.2)	
**Marital status**				
Married	455 (88.7)	137 (78.3)	592 (86.1)	0.002
Single	35 (6.8)	20 (11.4)	55 (8.0)	
Others	23 (4.5)	18 (10.3)	41 (6.0)	

P-value from Chi-square test; deviation from the grand total is due to missing data.

Patient socio-economic characteristics are presented in Table 2. More than 90% of the patients lived in public Housing and Development Board (HDB) flats, which is higher than that of the national population which is 84% [14]. Of those who provided their household income information, more than one third had monthly household income below S$2000, but this information should be interpreted with caution as almost one third of the interviewees’ household income was not reported. A substantial percentage of the patients (90%) had not obtained tertiary education.

**Table 2:** Patient socio-economic characteristics

**Characteristics**	**HbA1c ≤ 8.0%**	**HbA1c > 8%**	**Total**	**p-value**
	**(n = 513)**	**(n = 175)**	**(n = 688)**	
	**N (%)**	**N (%)**	**N (%)**	
**Occupation**				
Retired	198 (38.6)	42 (24.0)	240 (34.9)	0.001
Employed	160 (31.2)	78 (44.6)	238 (34.6)	
Unemployed	17 (3.3)	9 (5.1)	26 (3.8)	
Housewife	123 (24.0)	44 (25.1)	167 (24.3)	
Others	15 (2.9)	2 (1.1)	17 (2.5)	
**Housing type**				
Public housing 1–2 rooms	46 (9.1)	43 (7.4)	59 (8.7)	<0.001
Public housing 3 rooms	94 (18.5)	62 (35.4)	156 (22.9)	
Public housing 4 rooms	176 (34.7)	53 (30.3)	229 (33.6)	
Public housing 5 rooms or EC	152 (30.0)	30 (17.1)	182 (26.7)	
Private condominium / Landed property	39 (7.7)	17 (9.7)	56 (8.2)	
**Household income**				
SGD < $1000	115 (22.8)	34 (19.7)	149 (22.0)	0.333
SGD $1000-$1999	72 (14.3)	32 (18.5)	104 (15.3)	
SGD $2000-$3999	82 (16.2)	35 (20.2)	117 (17.3)	
SGD $4000-$5999	35 (6.9)	15 (8.7)	50 (7.4)	
SGD ≥ $6000	39 (7.7)	9 (5.2)	48 (7.1)	
Refuse to disclose/Unknown	162 (32.1)	48 (27.8)	210 (31.0)	
**Highest education**				
No formal education	156 (30.7)	50 (28.7)	206 (30.2)	0.792
Primary (PSLE)	142 (27.9)	53 (30.5)	195 (28.6)	
Secondary (O/N level)	130 (25.5)	43 (24.7)	173 (25.3)	
Post-secondary (A level)	25 (4.9)	5 (2.9)	30 (4.4)	
Post-secondary (ITE/NTC)	9 (1.8)	3 (1.7)	12 (1.8)	
Tertiary (Diploma/Degree)	47 (9.2)	20 (11.5)	67 (9.8)	

P-value from Chi-square test; deviation from the grand total is due to missing data.

Patient diabetic disease characteristics are presented in Table 3. Three-quarters (74.6%) of the patients were overweight or obese. Oral hypoglycaemic medication was the predominant regimen with 92.7% of patients receiving this treatment. Most of the patients (88.7%) complied with diabetes treatment most or all of the time. The most common co-morbidities in these diabetic patients are hypertension and hypercholesterolemia. Complications reported in this group of patients were micro-vascular conditions, including peripheral neuropathy (24.0%), retinopathy (24.5%), and kidney disease (7.7%), as well as macro-vascular conditions, including ischemic heart disease (13.9%), stroke (7.9%), and peripheral vascular disease (5.7%). It is interesting to note that over 90% of the patients were satisfied with the clinic management and were confident in their doctor.

**Table 3:** Patient diabetic disease characteristics

**Characteristics**	**HbA1c ≤ 8.0%**	**HbA1c > 8%**	**Total**	**p-value**
	**(n = 513)**	**(n = 175)**	**(n = 688)**	
	**N (%)**	**N (%)**	**N (%)**	
**BMI**				
<23 kg/m^2^	131 (27.0)	34 (20.9)	165 (25.4)	<0.001
23-27.4 kg/m^2^	228 (47.0)	55 (33.7)	283 (43.7)	
≥27.5 kg/m^2^	126 (26.0)	74 (45.4)	200 (30.9)	
**Smoking status**				
Never smoker	355 (69.5)	125 (71.8)	480 (70.1)	0.438
Ex-smoker	104 (20.4)	37 (21.3)	141 (20.6)	
Current smoker	52 (10.2)	12 (6.9)	64 (9.3)	
**Duration of DM**				
Less than 5 years	177 (34.6)	48 (27.4)	225 (32.8)	0.154
5-9.9 years	113 (22.1)	35 (20.0)	148 (21.6)	
10-14.9 years	89 (17.4)	31 (17.7)	120 (17.5)	
15-19.9 years	43 (8.4)	23 (13.1)	66 (9.6)	
20 years or above	89 (17.4)	38 (21.7)	127 (18.5)	
DM treatment*				
Insulin	34 (6.8)	39 (22.7)	73 (10.8)	<0.001
Medication	467 (91.6)	167 (96.0)	634 (92.7)	0.054
Diet controls	428 (84.4)	140 (80.5)	568 (83.4)	0.226
Exercise	314 (61.9)	106 (61.3)	420 (61.8)	0.877
**Compliance to medication**				
All the time	381 (74.6)	121 (69.1)	502 (73.2)	0.001
Most of the time	76 (14.9)	30 (17.1)	106 (15.5)	
Sometimes	15 (2.9)	17 (9.7)	32 (4.7)	
Rarely	0	1 (0.6)	1 (0.2)	
Never	1 (0.2)	1 (0.6)	2 (0.3)	
No medication is needed	38 (7.4)	5 (2.9)	43 (6.3)	
DM-related co-morbidities*				
Stroke	44 (8.6)	10 (5.8)	54 (7.9)	0.226
Ischaemic heart disease	75 (14.7)	20 (11.6)	95 (13.9)	0.302
Kidney disease	38 (7.4)	15 (8.6)	53 (7.7)	0.614
Peripheral neuropathy	122 (23.9)	42 (24.3)	164 (24.0)	0.925
Retinopathy	116 (22.8)	51 (29.7)	167 (24.5)	0.069
Peripheral vascular disease	26 (5.1)	13 (7.5)	39 (5.7)	0.249
Any of DM-related diseases	255 (49.9)	92 (52.9)	347 (50.7)	0.498
**Self-monitoring at home**	133 (26.0)	60 (34.5)	193 (28.2)	0.032
**Chronic medical problems**				
Hypertension	369 (72.2)	107 (61.5)	476 (69.5)	0.008
Hypercholesterolemia	367 (71.8)	125 (71.8)	492 (71.8)	0.996
History of cancer	21 (4.1)	8 (4.6)	29 (4.3)	0.790
Arthritis	163 (32.0)	51 (29.3)	214 (31.3)	0.515
Asthma	33 (6.5)	10 (5.8)	43 (6.3)	0.751
Lung disease	16 (3.1)	3 (1.7)	19 (2.8)	0.337
Liver disease	20 (3.9)	7 (4.1)	27 (4.0)	0.938
Mental disorders	26 (5.1)	6 (3.5)	32 (4.7)	0.383
Urology problems	53 (10.4)	14 (8.1)	67 (9.8)	0.383
ENT problems	45 (8.8)	10 (5.8)	55 (8.1)	0.210
Any of chronic medical problems	474 (92.8)	150 (86.2)	624 (91.1)	0.009
**Management satisfaction by clinic**				
Not satisfied	9 (1.8)	4 (2.3)	13 (1.9)	0.151
A little	35 (6.9)	21 (12.0)	56 (8.1)	
Mostly	297 (58.4)	91 (52.0)	388 (56.7)	
Very satisfied	168 (33.0)	59 (33.7)	227 (33.2)	
**Confidence in doctor**				
Not confident	5 (1.0)	1 (0.6)	6 (0.9)	0.280
A little	39 (7.7)	20 (11.5)	59 (8.6)	
Mostly	288 (56.6)	87 (50.0)	375 (54.9)	
Very confident	177 (34.8)	66 (37.9)	243 (35.6)	

P-value from Chi-square test; deviation from the grand total is due to missing data.

### Bivariate logistic regression analysis

Associations between patient characteristics and unacceptable HbA_1c_ control (>8%) by logistic regression analyses are presented in Table 4. The proportion of patients with unacceptable HbA_1c_ control was highest in the younger patient group (<60 years old) (36.6%) compared to the older patients groups (20.8% and 17.9%, respective). In bivariate logistic regression analysis, younger age is significantly associated with unacceptable HbA_1c_ control, with the OR being 0.45 and 0.38 respectively for the age bands of 60 to 69 years and 70 years and above, as compared to <60 years old. Within individual ethnic groups, a larger proportion of Malay (32.9%) and Indian (36.3%) diabetic patients had unacceptable HbA_1c_ control in comparison to Chinese diabetes patients (22.7%). The ORs (95% CI) of having unacceptable HbA_1c_ control for Malay and Indian versus Chinese patients were 1.68 (1.01 to 2.78) and 1.94 (1.18 to 3.20), respectively.

**Table 4:** Associations between patient characteristics by HbA1c levels by logistic regression analyses in type 2 diabetes mellitus patients treated and followed up in SingHealth polyclinics

**Variables**	**HbA1c**		**Unadjusted OR (95% CI)**	**Adjusted OR (95% CI)‡**
	**≤8.0%**	**≤8.0%**		
	**N (%)**	**N (%)**		
**Gender**				
Male	226 (74.2)	76 (25.8)	1.00 (ref)	1.00 (ref)
Female	285 (74.8)	99 (25.8)	1.03 (0.73-1.46)	1.20 (0.75-1.92)
**Age group**				
<60 years	156 (63.4)	90 (36.6)	1.00 (ref)	1.00 (ref)
60-69 years	187 (79.2)	49 (20.8)	0.45 (0.30-0.68)***	0.42 (0.25-0.73)**
≥ 70 years	165 (82.1)	36 (17.9)	0.38 (0.24-0.59)***	0.38 (0.20-0.73)**
**Ethnicity**				
Chinese	390 (77.4)	114 (22.6)	1.00 (ref)	1.00 (ref)
Malay	55 (67.1)	27 (32.9)	1.68 (1.01-2.78)*	1.02 (0.56-1.88)
Indian	51 (63.8)	29 (36.3)	1.94 (1.18-3.20)**	1.33 (0.72-2.45)
Others	17 (77.3)	5 (22.7)	1.00 (0.36-2.78)	0.50 (0.13-1.92)
**Marital status**				
Not married	58 (60.4)	38 (39.6)	1.00 (ref)	1.00 (ref)
Married	455 (76.9)	137 (23.1)	0.46 (0.29-0.72)***	0.59 (0.32-1.03)
**Smoking status**				
Never smoker	355 (74.0)	125 (26.0)	1.00 (ref)	/
Ex-smoker	104 (73.8)	37 (26.2)	1.01 (0.66-1.55)	/
Current smoker	52 (81.3)	12 (18.8)	0.66 (0.34-1.27)	/
**Occupation**				
Employed	160 (67.2)	78 (32.8)	1.00 (ref)	1.00 (ref)
Retired/Unemployed	215 (80.8)	51 (19.2)	0.49 (0.32-0.73)***	0.65 (0.36-1.16)
Housewife/Others	138 (75.0)	46 (25.0)	0.68 (0.44-1.05)	0.81 (0.43-1.49)
**Housing type**				
Public housing 1-3rooms	140 (65.1)	75 (34.9)	1.00 (ref)	1.00 (ref)
Public housing 4–5 rooms/Private	367 (78.6)	100 (21.4)	0.51 (0.36-0.73)***	0.52 (0.33-0.80)**
**Household income**				
SGD < $4000	269 (72.7)	101 (27.3)	1.00 (ref)	/
SGD ≥ $4000	74 (75.5)	24 (24.5)	0.86 (0.52-1.44)	/
**Highest education**				
No formal education	156 (75.7)	50 (24.3)	1.00 (ref)	/
PSLE/O/N level	272 (73.9)	96 (26.1)	1.10 (0.74-1.63)	/
A level/ITE/Tertiary	81 (74.3)	28 (25.7)	1.08 (0.63-1.84)	/
**Duration of DM**				
Less than 10 years	290 (77.8)	83 (22.2)	1.00 (ref)	1.00 (ref)
10 years or above	221 (70.6)	92 (29.4)	1.47 (1.04-2.08)*	1.73 (1.11-2.71)*
**DM treatment**				
Non-insulin	468 (77.9)	133 (22.1)	1.00 (ref)	1.00 (ref)
Insulin	34 (46.6)	39 (53.4)	4.03 (2.45-6.62)***	2.68 (1.45-4.95)**
**Compliance to medication**				
All the time/most of time	455 (75.1)	151 (24.9)	1.00 (ref)	1.00 (ref)
Less than all the time	16 (45.7)	19 (54.3)	3.59 (1.80-7.51)***	3.72 (1.55-7.64)***
**Self-monitoring**				
No	378 (76.8)	114 (23.2)	1.00 (ref)	1.00 (ref)
Yes	133 (68.9)	60 (31.1)	1.50 (1.03-2.17)*	1.27 (0.79-2.02)
**BMI**				
<23 kg/m^2^	130 (79.3)	34 (20.7)	1.00 (ref)	1.00 (ref)
23-27.4 kg/m^2^	227 (80.5)	55 (19.5)	0.93 (0.57-1.50)	0.91 (0.53-1.55)
≥27.5 kg/m^2^	173 (70.0)	74 (30.0)	2.25 (1.40-3.61)***	1.78 (1.01-3.13)
**Management satisfaction by clinic**				
Very satisfied	168 (74.0)	59 (26.0)	1.00 (ref)	/
Mostly	297 (76.6)	91 (23.5)	0.87 (0.60-1.27)	/
Not satisfied/A little	44 (63.8)	25 (36.2)	1.62 (0.91-2.87)	/
**Confidence in doctor**				
Very confident	177 (72.8)	66 (27.2)	1.00 (ref)	/
Mostly	288 (76.8)	87 (23.2)	0.81 (0.56-1.17)	/
A little/Not confident	44 (67.7)	21 (32.3)	1.28 (0.71-2.31)	/

*P-value < 0.05; **p-value < 0.01; ***p-value < 0.001.

‡Multivariate logistic regression model, adjusted for age (<60, 60–69, ≥70 years), gender (male, female), race (Chinese, Malay, Indian, Others), marital status (married, single, others), occupation (employed, retired/unemployed, housewife/others), housing type (public1-4, public5/private), BMI (<23, 23–27.5, >27.5), duration of DM (<10, ≥10 years), DM treatment (non-insulin, insulin), compliance to medication (all the time, less than most of time), monitor glucose at home (yes, no).

The proportion of patients with unacceptable HbA_1c_ control increased with the duration of diabetes in the studied patient population. The proportion was 22.2% in patients with less than 10 years of DM history, but increased to 29.4% in patients with 10 or more years of DM history. The OR of having unacceptable HbA_1c_ control for patients with ≥ 10 years of DM versus those had DM for <10 years was 1.47 (95% CI: 1.04 to 2.08).

Differences in the percentage of patients with unacceptable HbA_1c_ control in terms of different treatment regimens were observed with a higher rate in patients receiving insulin (53.4%) and lower in the patients who were not on insulin (22.1%). The risk was also significantly higher in the insulin patient group with OR of 4.03 (95% CI: 2.45 to 6.62), showing the treatment regimens corresponding to the severity of disease.

Patient’s compliance to medication treatment is critical in the management of diabetes mellitus. A large proportion of patients (54.3%) whose self-reported compliance to medication was less optimal had unacceptable HbA_1c_ control than patients (24.1%) who at least complied with medication treatment most of the time. The OR of having unacceptable HbA_1c_ control was 3.59 (95% CI: 1.80 to 7.51) for patients who had suboptimal compliance to medication as compared to patients who complied with medication treatment.

Obesity is a major risk factor for developing diabetes mellitus in adults. In the study population, more obese patients (30.0%) had unacceptable HbA_1c_ control compared to diabetes patients with normal BMI (20.7%) and the OR of unacceptable HbA_1c_ control for obesity versus normal weight was 2.25 (95% CI: 1.40- 3.61).

Patients who were not married, living in smaller public housing flats (≤3 rooms), or carrying out self-glucose monitoring at home were also more likely to have unacceptable glycaemic control. Other factors, such as gender, smoking history, household income, education level, satisfaction with clinic and confidence on the doctor were not associated with unacceptable HbA_1c_ control.

### Multivariate logistic regression analysis

Multivariate logistic regression analysis is presented in Table 4. Statistically significant variables associated with unacceptable HbA_1c_ control identified from the bivariate logistic regression analyses and well known confounding factors for glycaemic control including age, gender and ethnicity were included in the multivariate logistic regression model. The other variables included marital status, occupation status, housing type, duration of diabetes, type of diabetes treatment, compliance to medication treatment, BMI, and self-monitoring of glucose at home. All complications and co-morbidities were not significant in the bivariate logistic regression analysis, including hypertension, hypercholesterolemia, stroke, ischemic heart disease, kidney disease, peripheral neuropathy, retinopathy, and peripheral vascular disease, and have not been included in Table 4.

In this analysis, statistical significance was observed for age older than 60 years old versus <60 years old (OR = 0.42 for 60–69 years [95% CI: 0.25 to 0.73] and 0.38 for ≥70 years [95% CI: 0.20 to 0.73]) , living in larger housing versus 1–3 room public housing (OR = 0.52, 95% CI: 0.33 to 0.80), ≥10 years of diabetes history versus <10 years of DM (OR = 1.73, 95% CI: 1.11 to 2.71) , presence versus absence of insulin treatment for diabetic control (OR = 2.68, 95% CI: 1.45 to 4.95), and poor versus good compliance to medication (OR = 3.72, 95% CI: 1.55 to 7.64). Not being married and obesity were borderline significantly associated with unacceptable glycaemic control.

## Discussion

In our study, the mean HbA_1c_ level was 7.6% and the median HbA1c was 7.3%, and 25.4% of patients had an unacceptable HbA1c (>8%). This result is comparable with studies conducted in the US [15] where the mean HbA1c was 7.6% and 37.1% had HbA1c >8%, as well as in Australia [16] where 24.3% of patients had HbA1c equal or more than 8.0%. A study conducted in another local polyclinic in 2003 [7] observed a mean HbA_1c_ of 8.3%, while the Chronic disease management plan (CDMP) 2007 in Singapore showed that 31.1% of patients had HbA_1c_ >8% [17].

Younger patients (<60 years old) in our study had poorer glycaemic control. In the literature, the evidence for the association between age and glycaemic control in type 2 diabetic patients is mixed. Some studies have found that glycaemic control is better in younger patients [18], while others have shown no effect [19,20]. Several studies in Singapore [7], United States [21,22], Netherlands [6] and Germany [23] have similar findings with our study, which have also shown that younger patients have poorer glycaemic control.

Recent evidence suggests that early-onset type 2 diabetes mellitus is a more aggressive disease phenotype than the later-onset cohort, and these early-onset diabetics experience high complication burden [24].

Health is a value, and to some it may not be the highest value [25]. Younger adult patients may be less motivated to manage their diabetic condition, as they may be busy with their job, and have less time to comply with a healthy lifestyle, medication and clinic visits. A patient with early diabetes prior to the onset of complications and asymptomatic, in whom the quality of life has not yet been affected, may not perceive the need for good diabetic control [12].

In our study, we have also analyzed the characteristics peculiar to the younger group of diabetic patients that could have contributed to the poorer control. Younger adult diabetics were more likely to be employed, unmarried and better educated. Diabetics who were obese, smoking, less confident in their doctor, and less adherent to medication were also younger. This is of public health importance, and targeting their lifestyle and behavioural factors could be the key to better glycaemic control in this group of patients.

Effective educational and behaviour modification programmes would be required to target younger diabetics, as this group with earlier disease onset may have a longer life expectancy relevant to prevention of complications. Evidence has shown that good glycaemic control may be beneficial especially earlier in the disease course [26], and benefits emerge in the long term [27]. This phenomenon presents major governmental, societal, cultural, public health and medical challenges to promote healthy lifestyle in the early years and to administer timely optimized medical care to prevent or reduce the onset of complications in these diabetic patients.

Longer duration of diabetes was associated with poorer HbA_1c_ control. This observation is consistent with other studies [6,21]. This reflects the natural progression of type 2 diabetes mellitus due to progressive pancreatic beta cell failure.

Patients using insulin in their treatment regimen had a higher rate of suboptimal glycaemic control (53.4%) compared to patients without insulin treatment (22.1%). This finding is consistent with other studies [7,20,24]. This observation is likely due to the fact that in the current guidelines, the indication for starting patients on insulin is when their diabetic control is not optimally controlled by oral medication, and hence these patients on insulin are patients with advanced diabetes where good glycaemic control is less likely to be achieved. Difficulty of maintaining glycaemic control while minimizing hypoglycemia, weight gain as an anabolic effect of insulin, and non-compliance with diet, can also contribute to poorer control with insulin.

Being unmarried, as well as obesity, were borderline significantly associated with unacceptable diabetic control. A stable life partner could potentially provide a strong social and emotional support for a patient with chronic disease like diabetes, to aid the patient in maintaining a healthy lifestyle and compliance to treatment. Obesity is also a well-known factor associated with poorer glycaemic control [28].

Our study demonstrated that better self-reported compliance to medication treatment is associated with better HbA_1c_ control. This knowledge is beneficial for the healthcare provider and patients in diabetes management to encourage adherence to therapy.

The strengths of the study include: this study was the first large scale study conducted for type 2 diabetes patients who are treated and followed up in all SingHealth Polyclinics; patient recruitment was conducted in eight SingHealth Polyclinics with response rate of 72.5% which is reasonable for survey studies; the study population is representative of diabetes patients seeking medical treatment in the polyclinics; the study captured a broad range of data which provide an important insight into possible predictors associated with poor glycaemic control in diabetes patients in primary care.

The limitations in this study include: firstly, the cross-sectional study would not be able to establish causal-effect relationship of the significant factors identified. Secondly, all variables other than HbA_1c_ were based on self-reporting. There were no methods in place to verify the accuracy or reliability of the data collected through questionnaire survey.

Increased attention should be paid to patients who are younger, patients who have had longer diabetes history, patients who are on insulin therapy and patients who are less compliant to medication treatment. The challenge is to develop the most effective strategy to continually enhance diabetes management for our diabetic patients in primary care.

## Conclusions

Treating type 2 diabetic patients to target is an ongoing challenge. Younger adult patients were found to have poorer glycaemic control, and hence targeted educational and behaviour modification programmes would be required to effectively manage this group of younger diabetics.

## Competing interests

The authors declare that they have no competing interests.

## Authors’ contributions

HMJQ was responsible for conducting the literature review, designing the study, and writing the manuscript. YPL was responsible for performing the statistical analysis, interpreting the data and drafting the manuscript. NL was responsible for designing the study, supervising the conduct of the surveys, interpreting the data, and revising the manuscript. CHH was responsible for conceptualizing and revising the manuscript. EGT was overseeing the study. All authors have read and approved the final manuscript.

## Pre-publication history

The pre-publication history for this paper can be accessed here:

http://www.biomedcentral.com/1472-6823/13/18/prepub
